# Formative vs. summative assessment: impacts on academic motivation, attitude toward learning, test anxiety, and self-regulation skill

**DOI:** 10.1186/s40468-022-00191-4

**Published:** 2022-09-13

**Authors:** Seyed M. Ismail, D. R. Rahul, Indrajit Patra, Ehsan Rezvani

**Affiliations:** 1 College of Humanities and Sciences, Prince Sattam Bin Abdulaziz University, Al-Kharj, Saudi Arabia; 2 School of Science and Humanities, Shiv Nadar University Chennai, Chennai, India; 3 NIT Durgapur, Durgapur, West Bengal India; 4 English Department, Isfahan (Khorasgan) Branch, Islamic Azad University, Isfahan, Iran

**Keywords:** Academic motivation, Attitude toward learning, Formative assessment, Self-regulation skill, Summative assessment, Test anxiety

## Abstract

As assessment plays an important role in the process of teaching and learning, this research explored the impacts of formative and summative assessments on academic motivation, attitude toward learning, test anxiety, and self-regulation skill of EFL students in Iran. To fulfill the objectives of this research, 72 Iranian EFL learners were chosen based on the convenience sampling method assigned to two experimental groups (summative group and formative group) and a control group. Then, the groups took the pre-tests of test anxiety, motivation, and self-regulation skill. Then, one experimental group was trained by following the rules of the formative assessment and the other experimental group was taught according to the summative assessment. The control group was instructed without using any preplanned assessment. After a 15-session treatment, the post-tests of the test anxiety, motivation, and self-regulation skill were administered to all groups to assess the impacts of the instruction on their language achievement. Lastly, a questionnaire of attitude was administered to both experimental groups to examine their attitudes towards the impacts of formative and summative assessment on their English learning improvement. The outcomes of one-way ANOVA and Bonferroni tests revealed that both summative and formative assessments were effective but the formative one was more effective on academic motivation, test anxiety, and self-regulation skill. The findings of one sample *t*-test indicated that the participants had positive attitudes towards summative and formative assessments. Based on the results, it can be concluded that formative assessment is an essential part of teaching that should be used in EFL instructional contexts. The implications of this study can help students to detect their own weaknesses and target areas that need more effort and work.

## Introduction

In teaching and learning, assessment is defined as a procedure applied by instructors and students during instruction through which teachers provide necessary feedbacks to modify ongoing learning and teaching to develop learners’ attainment of planned instructional aims (Robinowitz, 2010). According to Popham (2008), assessment is an intended procedure in which evidence of learners’ status is utilized by educators to adjust their ongoing instructional processes or applied by learners to change their present instructional strategies. Assessment intends to improve learning and it is used to reduce the gap between students’ present instructional situation and their target learning objectives (Heritage, 2012).

Two types of assessment are formative and summative. According to Glazer (2014), summative assessment is generally applied to give learners a numerical score with limited feedback. Therefore, summative assessment is commonly used to measure learning and is rarely used for learning. Educators can make the summative assessment more formative by giving learners the opportunity to learn from exams. This would mean supplying pupils with feedback on exams and making use of the teaching potentiality of exams. Wininger (2005) proposed an amalgamation of assessment techniques between summative assessment and formative assessment. This marriage between summative assessment and formative assessment is referred to as summative-formative assessment. Based on Wininger, summative-formative assessment is used to review the exam with examinees so they can get feedback on comprehension. Formative-summative assessment occurs in two primary forms: using a mock exam before the final or using the final exam before the retake.

Formative assessment allows for feedback which improves learning while summative assessment measures learning. Formative assessment refers to frequent, interactive assessments of students’ development and understanding to recognize their needs and adjust teaching appropriately (Alahmadi et al., 2019). According to Glazer (2014), formative assessment is generally defined as tasks that allow pupils to receive feedback on their performance during the course. In the classroom, teachers use assessments as a diagnostic tool at the termination of lessons or the termination of units. In addition, teachers can use assessments for teaching, by identifying student misconceptions and bridging gaps in learning through meaningful feedback (Dixson & Worrell, 2016). Unfortunately, numerous instructors consider formative assessments as a tool to measure students’ learning, while missing out on its teaching potential. Testing and teaching can be one or the same which will be discussed further in this research (Remmi & Hashim, 2021).

According to Black et al. (2004), using formative tests for formative purposes improves classroom practice whereby students can be encouraged in both reflective and active review of course content. In general terms, formative assessment is concerned with helping students to develop their learning (Buyukkarci & Sahinkarakas, 2021). Formative assessment can be considered as a pivotal and valid part of the blending of assessment and teaching (Ozan & Kıncal, 2018). Formative assessment helps students gain an understanding of the assessment process and provides them with feedback on how to refine their efforts for improvement. However, in practice, assessment for learning is still in its infancy, and many instructors still struggle with providing productive and timely feedback (Clark, 2011).

Using the mentioned assessments can positively affect the test anxiety of the students. Test anxiety signifies the extent to which the students experience apprehension, fear, uneasiness, panic tension, and restlessness while even thinking of forthcoming tests or exams (Ahmad, 2012). Anxiety can also be regarded as a product of hesitation about imminent events or situations (Craig et al., 2000). Test anxiety is the emotional reaction or status of stress that happens before exams and remains throughout the period of the exams (Sepehrian, 2013). Anxiety can commonly be connected to coercions to self-efficacy and evaluations of circumstances as threatening or reactions to a resource of stress to continue (Pappamihiel, 2002).

The other variable which can influence the consequences of tests or testing sessions in EFL settings is the attitudes of students towards English culture, English language, and English people. Kara (2009) stated that attitude about learning together with beliefs and opinions have a significant impact on learners’ behaviors and consequently on their performances. Those learners who have desirable beliefs about language learning are willing to rise more positive attitudes toward language learning. On the other hand, having undesirable beliefs can result in negative attitudes, class anxiety, and low cognitive achievements (Chalak & Kassaian, 2010; Tella et al., 2010). There are both negative and positive attitudes towards learning. Positive attitudes can develop learning and negative attitudes can become barriers to learning because students have these attitudes as they have difficulties in learning or they just feel that what is presented to them is boring. While a negative attitude toward learning can lead to poor performances of students, a positive attitude can result in appropriate and good performances of students (Ellis, 1994).

Woods (2015) says that instructors should regularly utilize formative assessment to advance the learners’ self-regulation skills and boost their motivation. Motivation is referred to the reasons why people have different behaviors in different situations. Motivation is considered as the intensity and direction of the students’ attempts. The intensity of attempt is referred to the extent that students try to reach their objectives and the direction of attempt is referred to the objectives that students intend to reach (Ahmadi et al., 2009; Paul & Elder, 2013). Motivation is an inborn phenomenon that is influenced by four agents such as aim (the aim of behaviors, purposes, and tendencies), instrument (instruments used to reach objectives), situation (environmental and outer stimulants), and temper (inner state of the organism). To reach their goals, people first should acquire the essential incentives. For instance, academic accomplishment motivation is significant to scholars (Firouznia et al., 2009).

Wiliam (2014) also asserts that self-regulation learning can be a crucial part of a productive formative assessment concerning the techniques of explaining, sharing, and understanding the instructional goals and students’ success and responsibility for their own learning. Self-regulation skill requires learners to dynamically utilize their cognitive skills; try to achieve their learning aims; receive support from their classmates, parents, and instructors when needed; and most significantly, be responsible for their own learning (Ozan & Kıncal, 2018). This research aimed to explore the impacts of using summative and formative assessments of Iranian EFL learners’ academic motivation, attitude toward learning, test anxiety, and self-regulation skill. This study is significant as it compared the effects of two kinds of assessments namely formative and summative on academic motivation, attitude toward learning, test anxiety, and self-regulation skill. As this research investigated the effects of the mentioned assessments on four emotional variables simultaneously, it can be considered as a novel study.

## Review of the literature

In the field of teaching English as a foreign language, several researchers and experts defined the term “assessment” as a pivotal component of the process of teaching. According to Brown (2003), assessment is a process of collecting data about learners’ capabilities to conduct learning tasks. That is, assessment is the way instructors use to gather data about their methods and their pupils’ improvement. Furthermore, the assessment process has got an inseparable component from teaching, since it is impossible to think of teaching without assessments. Brown (2003) defined assessment in relation to testing. The differences between them refer to the fact that the latter occurs at an identified point of time while the former is an ongoing process that occurs regularly (Brown, 2003).

Other scholars explained the meaning of assessment by distinguishing it from evaluation. Regarding the difference between the two, Nunan (1992) asserted that assessment is referred to the procedures and processes whereby teachers determine what students can do in the target language and added evaluation is referred to a wider range of processes that may or may not include assessment data. In this way, then, assessment is process-oriented while evaluation is product-oriented. Palomba and Banta (1999) defined assessment as “the systematic collection, review, and use of information about educational programs undertaken to improve learning and development” (p.4). All in all, assessing students’ performances means recognizing and gathering information, receiving feedback, and analyzing and modifying the learning processes. The main goal, thus, is to overcome barriers to learning. Assessment is then used to interpret the performances of students, develop learning, and modify teaching (Aouine, 2011; Ghahderijani et al., 2021).

Two types of assessment are formative and summative. Popham (2008) said that it is not the nature of the tests to be labeled as summative or formative but the use to which that tests’ outcomes will be put. That is to say, the summative-formative manifestation of assessment does not stop at being a typology but it expands to be purposive due to the nature of assessment. Summative assessment, then, has been referred to as some criteria. Cizek (2010) suggests that two criteria can define the summative assessment: (1) it is conducted at the termination of some units and (2) its goal is mainly to characterize the performances of the students or systems. Its major goal is to gain measurement of attainment to be utilized in making decisions.

Through Cizek’s definition, a summative assessment seeks to judge the learners’ performances in every single course. Thus, providing diagnostic information is not what this type of assessment is concerned with. Significantly, the judgments made about the students, teachers, or curricula are meant to grade, certificate, evaluate, and research on how effective curricula are, and these are the purposes of summative assessment according to Cizek (2010).

According to Black and Wiliam (2006), summative assessment is given occasionally to assess what pupils know and do not know. This type of assessment is done after the learning has been finalized and provides feedback and information that summarize the learning and teaching process. Typically, no more formal learning is occurring at this stage, other than incidental learning that may happen via completing the assignments and projects (Wuest & Fisette, 2012). Summative assessment measures what students have learned and mostly is conducted at the end of a course of instruction (Abeywickrama & Brown, 2010; Liu et al., 2021; Rezai et al., 2022).

For Woods (2015), the summative assessment provides information to judge the general values of the instructional programs, while the outcomes of formative assessment are used to facilitate the instructional programs. Based on Shepard (2006), a summative assessment must accomplish its major purpose of documenting what learners know and can do but, if carefully created, should also efficaciously fulfill a secondary objective of learning support.

Brown (2003) claimed that summative assessment aims at measuring or summarizing what students have learned. This means looking back and taking stock of how well that students have fulfilled goals but does not essentially pave the way to future improvement. Furthermore, the summative assessment also known as assessment of learning is clarified by Spolsky and Halt (2008) who state that assessment of learning is less detailed, and intends to find out the educational programs or students’ outcomes. Thus, summative assessment is applied to evaluating different language skills and learners’ achievements. Even though summative assessment has a main role in the learners’ evaluation, it is not sufficient to know their advancement and to detect the major areas of weaknesses, and this is the essence of formative assessment (Pinchok & Brandt, 2009; Vadivel et al., 2021).

The term ‘formative assessment’ has been proposed for years and defined by many researchers. A clearer definition is provided by Brown (2003) in which he claims that formative assessment is referred to the evaluation of learners in the process of “forming” their skills and competencies to help them to keep up that growth process. It is also described as comprising all those activities conducted by instructors or by their learners that supply information to be utilized as feedback to adjust the learning and teaching activities in which they are involved (Fox et al., 2016).

Formative assessments aim to gain immediate feedback on students learning through which strengths and weaknesses of students can be diagnosed. Comprehensively, Wiliam (2011) suggests: Practices in the classrooms are formative to the extent that evidence about students’ accomplishments is elicited, interpreted, and utilized by instructors, students, or their classmates, to decide about the subsequent steps in the education that are probably to be better or better founded, than the decisions they would have taken in the absence of the evidence that was elicited.

Through this definition, formative assessment actively involves both students’ and teachers’ participation as a key component to develop students’ performance. The assessment for learning, which is based on the aim behind using it, is assessing learners’ progress (McCallum & Milner, 2021). Therefore, it is all about gathering data about learners’ achievement to recognize their progress in skills, requirements, and capabilities as their weaknesses and strengths before, during, and after the educational courses to develop students’ learning and achievement (Douglas & Wren, 2008).

Besides, Popham (2008) considered the formative assessment as a strategic procedure in which educators or pupils utilize assessment-based evidence to modify what they are presently performing. That describes it as the planned process that is not randomly occurring. Therefore, formative assessment is an ongoing procedure that provides learners with constructive timely feedback, helping them achieve their learning goals and enhancing their achievements (Vogt et al., 2020). Formative assessment is a helpful technique that can provide students with formative help by evaluating the interactions between assessment and learning (Chan, 2021; Masita & Fitri, 2020).

Some criteria related to formative assessment have been presented by Cizek (2010). In his opinion, formative assessment attempts to identify students’ levels whether high or low, to provide more help for educators to plan subsequent instruction, to make it easier for students to continue their own learning, review their work, and be able to evaluate themselves. To make learners responsible for their learning and do their research Formative assessment, to Cizek, is a sufficient tool and area for learners and teachers to make proficiency in the learning-teaching process. All in all, concerning specific objectives, formative assessment is a goal-oriented process.

Tahir et al. (2012) stated that formative assessment is a diagnostic use of assessment that can provide feedback to instructors and learners throughout the instructional process. Marsh (2007) claimed that formative tests are a type of strategy which are prepared to recognize students’ learning problems to provide a remedial procedure to develop the performances of the majority of the learners. The information that is provided for the learners should be utilized for the assessment to be explained as a formative one. The Assessment Reform Group (ARG) (2007) explains formative assessment as the procedure to look for and interpret the evidence for instructors and their students to make decisions about where the students fit in their learning, where they need to go, and how best to get there. Kathy (2013) also argued that formative tests aim to analyze the students’ learning problems to develop their academic attainment.

The theory that is behind our study is the sociocultural theory stating that knowledge is generated in a cooperative way within social contexts. It views learning as a condition wherein learners generate their meanings from the materials and content delivered to them, rather than trying to memorize the information (Vygotsky, 1978). Based on sociocultural theory, learning can occur successfully when teachers and students have more interactions with each other.

Some empirical studies are reported here. Alahmadi et al. (2019) aimed to examine whether a formative speaking assessment produced any effect on learners’ performances in the summative test. Besides, they aimed to observe students’ learning and to provide useful feedbacks that can be applied by educators to develop learners’ achievement and assist them to detect their weaknesses and strengths in speaking skills. Their results indicated that formative assessment helped Saudi learners to solve the problems they encounter in speaking tests.

Mahshanian et al. (2019) highlighted the significance of summative assessment in conjunction with teacher-based (formative) assessments on the learners’ performances. To do this study, 170 EFL students at the advanced level were chosen and grouped based on the kind of assessment they had received. The subjects in this research were administered exams for two main reasons. First, a general proficiency test was given to put the students at different levels of proficiency. Second, for comparing students’ development according to different kinds of assessments within a 4-month learning duration, an achievement test of the course was administered both as the pre-test and the post-test. The data gained via the scores of the participants on the achievement test received analyses and then compared by utilizing ANCOVA, ANOVA, and *t-*tests. Based on the outcomes of this research, we can conclude that an amalgamation of summative and formative assessments can result in better achievements for EFL students than either summative or formative assessments discretely.

Imen (2020) attempted to determine the effects of formative assessments on EFL learners’ writing skills. Indeed, the goal of this study was to recognize the effects of formative assessments on developing the writing skills of first-year master’s students at Abdel Elhamid Ibn Badis University, in Mostaganem. This research also attempted to reveal an essential issue that is the lack of the execution of formative assessments in the writing classrooms. To verify the hypotheses, two tools were applied in this study to gather the data, the teachers’ questionnaire and the students’ questionnaire. The findings of the study revealed that the formative assessment was not extensively used in teaching and learning writing skills, at the University of Mostaganem. The results of both questionnaires showed that if the students were evaluated formatively, their writing skills could be highly enhanced.

Ashdale (2020) attempted to examine the influences of a particular formative assessment named as Progress Trackers, by comparing a control group that did not receive the Progress Tracker with an experimental group that received the formative-based assessment. The research findings revealed that there were no substantial differences between the experimental and control groups based on the results of the pre-test and the post-test scores. While not statistically significant, the experimental group showed a larger increase in the learners with at least a 60% development in achievement. The lack of significant differences between the experimental group and the control group could be created by the uselessness of the formative assessments or the inability to exclude other factors in the class contexts. This could comprise the uses of other formative assessments applied in both groups, delivery of content, and execution of the formative assessments.

Persaud Singh and Ewert (2021) investigated the effects of quizzes and mock exams as a formative assessment on working adult learners’ achievement using a quasi-experimental quantitative design. One experimental group received both quizzes and mock exams, another group received mock exams only, and a control group received neither. The data gathered received analyses by utilizing *t*-tests and ANOVA. The findings indicated noticeable differences in the levels of achievement for the groups receiving formative assessments in comparison to the control participants. The “mock exam” group outperformed slightly than the “quizzes and mock exam” group.

Al Tayib Umar and Abdulmlik Ameen (2021) traced the effects of formative assessment on Saudi EFL students’ achievement in medical English. The research also tried to figure out teachers’ and students’ attitudes toward formative assessment. The participants involved in this research were 98 students selected among the Preparatory Year learners at a Saudi university. They were assigned to an experimental group and a control group. The experimental students were given their English for Specific Purposes (ESP) courses following the formative assessment techniques whereas the control group was trained in their ESP courses by traditional assessment rules. The experimental group teachers were given intensive training courses in Saudi Arabia and abroad on how to use formative assessment principles in the classrooms. At the end of the experiment that continued for 120 days, the control and experimental groups sat for the end of term examination which was designed for all candidates in the Preparatory College. Grades of all participants in the two groups in the final exam were compared. The performance of the experimental group was found to be meaningfully higher than that of the control group. Instructors’ and students’ attitudes towards formative assessment were positive.

Hamedi et al. (2022) investigated the effects of using formative assessment by Kahoot application on Iranian EFL students’ vocabulary knowledge as well as their burnout levels. This study was conducted on 60 participants who were in two groups of experimental and control. The results indicated that using formative assessment generated significant effects on of Iranian EFL students’ vocabulary knowledge.

In conclusion, the above studies confirmed the positive effects of summative and formative assessment on language learning. Yet, there are a few kinds of research on comparing the effects of the summative and formative assessments on Iranian EFL learners’ academic motivation, attitude toward learning, test anxiety, and self-regulation skill. Most studies in the domain of assessment examined the effects of the summative and formative assessments on the main skills (reading, speaking, writing, and listening) and they did not pay much attention to the psychosocial variables; therefore, this research posed two questions to cover the existing gap.

- RQ1. Does using formative and summative assessments positively affect Iranian EFL learners’ test anxiety, academic motivation, and self-regulation skill?
- RQ2. Do Iranian EFL learners present positive attitudes toward learning through formative and summative assessments?

## Methodology

### Design of the study

#### Participants

The participants of this research were 72 Iranian EFL students who have studied English since 2016. The male EFL learners were selected based on the convenience sampling method by administering the Preliminary English Test (PET). They were selected from the Parsian English language institute, located in Ahvaz city, Iran. The participants’ general English proficiency was intermediate and their age average was 21 years old. The participants were divided into two experimental groups (summative and formative) and a control group.

#### Instrumentations

For homogenizing the subjects in terms of general English proficiency, we gave a version of the PET test, extracted from the book *PET Practice Test* (Quintana, 2008). Because of some limitations, only the sections of reading, grammar, and vocabulary of the test were used in this study. We piloted the test on another similar group and allotted 60 min for answering all its items. Its validity was accepted by some English experts and its reliability was .91.

Britner and Pajares’ (2006) Science Anxiety Scale (SAS) was used as the other instrument to assess the participants’ test anxiety. Some wordings of the items were changed to make them suitable for measuring test anxiety. There were 12 items in this test that required the participants to consider the items (e.g., I am worried that I will get weak scores in most of the exams) and answer a 6-point scale ranging from certainly false to certainly true. Based on Cronbach’s alpha formula, the reliability index of the anxiety test was .79.

The other tool used in this study was the Self-Regulatory Strategies Scale (SRSS) which was developed by Kadıoğlu et al. (2011) to assess the self-regulation skills of the participants. The SRSS was a 6-point Likert instrument including never, seldom, occasionally, often, frequently, and constantly. The SRSS consisted of 29 statements in eight dimensions. The results of Cronbach’s alpha formula showed that the reliability of the SRSS was .82.

We used the Attitude/Motivation Test Battery (AMTB) of Gardner (2004) to evaluate the respondents’ English learning motivation. This measuring instrument had 26 items each with six responses: Highly Disagree, Moderately Disagree, Somewhat Disagree, Somewhat Agree, Moderately Agree, and Highly Agree. We used the Cronbach alpha to measure the reliability of the motivation questionnaire (*r* = .87). It should be noted that the motivation questionnaire, the SAS, and the SRSS were used as the pre-tests and post-tests of the research.

The last tool employed in this research was an attitude questionnaire examining the participants’ attitudes towards the effectiveness of summative and formative assessment on their English learning enhancement. The researchers themselves created 17-point Likert- items for this questionnaire and the reliability of this instrument was .80. Likert scale was utilized in the questionnaire to show the amount of disagreement and agreement from 1 to 5 that were highly disagree, disagree, no idea, agree, and highly agree. The validities of all mentioned tools were substantiated by a group of English specialists.

#### Collecting the needed data

To start the study, first, the PET was administered to 96 EFL learners and 72 intermediate participants were selected among them. As stated previously, the participants were divided into two experimental groups (summative and formative) and one control group. After that, the pretests of test anxiety, motivation, and self-regulation skill were administered to the participants of all groups. After pretesting process, the treatment was conducted on the groups differently; each group received special instruction.

One experimental group was instructed based on the rules of the formative assessment, in the formative group, the teacher (researcher) assisted the students to participate in evaluating their learning via using self and peer assessment. Besides, the teacher’s comprehensive and descriptive elicitation and feedbacks of information about students’ learning were significant in formative class. In fact, there were no tests at the termination of the term and the teacher was flexible concerning the students’ mistakes and provided them with constructive feedback including metalinguistic clues, elicitation, correction, repletion, clarification request, recast, and repletion.

In the summative class, the teacher assessed the students’ learning by giving mid-term and final exams. The teacher did not provide any elaborative feedback, and his feedback was limited to yes/no and true/ false. The control group neither received a formative-based instruction nor a summative-based instruction. The teacher of the control group instructed them without utilizing any preplanned assessments. They finished the course without any formative and summative assessments. After the treatment, the post-tests of the test anxiety, motivation, and self-regulation skill were given to all groups to assess the influences of the intervention on their language achievement. In the final step, the questionnaire of attitude was distributed among both experimental groups to check their opinions about the impacts of summative and formative assessment on their English learning improvement.

The whole study lasted 23 sessions; each took 50 min. In one session, the PET test was administered and in the next three sessions, three pre-tests were conducted. During 15 sessions, the treatment was carried out; in three sessions, three post-tests were given to the participants, and in the last session the attitudinal questionnaire was administered to examine the participants’ attitudes towards the effectiveness of summative and formative assessment of their English learning achievement.

#### Data analysis

Having prepared all needed data via the procedures mentioned above, some statistical steps were taken to provide answers to the questions raised in this study. First, the data were analyzed descriptively to compute the means of the groups. Second, some one-way ANOVA and Bonferroni tests were used for analyzing the data inferentially. Third, one sample *t-*test was utilized to analyze the motivation questionnaire data.

## Results and discussion

After checking and getting sure about the normality distribution of the data by using the Kolmogorov-Smirnov test, we used several one-way ANOVA tests and reported their results in the following tables:

As we see in Table 1, the mean scores of all groups are almost similar. They got almost equal scores on their anxiety pre-test and the three groups were at the same level of anxiety before conducting the instruction. This claim is verified in the following table with the help of one-way ANOVA.

**Table 1:** Descriptive statistics of all groups on the test anxiety pre-tests

	*N*	Means	Std. deviations	Std. errors	95% confidence interval for means		Minimum	Maximum
					Lower bounds	Upper bounds		
Control	24	27.70	11.37	2.32	22.90	32.51	14.00	49.00
Summative	24	28.91	11.89	2.42	23.89	33.93	13.00	50.00
Formative	24	28.41	10.93	2.23	23.79	33.03	14.00	49.00
Total	72	28.34	11.25	1.32	25.70	30.99	13.00	50.00

According to the Sig value in Table 2, there is not a noticeable difference between the test anxiety of all three groups. They were at the same anxiety level at the outset of the study. The inferential statistics show that all the participants had an equal amount of anxiety before they had received the treatment.

**Table 2:** Inferential statistics of all groups on the test anxiety pre-tests

	Sum of squares	df	Mean squares	*F*	Sig.
Between groups	17.69	2	8.84	.06	.93
Within groups	8980.62	69	130.15		
Total	8998.31	71			

As is seen in Table 3, the mean scores of all groups are different on the anxiety post-tests. Based on the descriptive statistics, the groups gained different scores on their anxiety post-test and the experimental groups obtained better scores than the control group. This claim is substantiated in the following table by using a one-way ANOVA test.

**Table 3:** Descriptive statistics of all groups on the test anxiety post-tests

	*N*	Means	Std. deviations	Std. errors	95% confidence interval for means		Minimum	Maximum
					Lower bounds	Upper bounds		
Control	24	29.95	11.08	2.26	25.27	34.63	14.00	51.00
Summative	24	37.91	10.80	2.20	33.35	42.47	19.00	60.00
Formative	24	49.50	10.37	2.11	45.11	53.88	23.00	62.00
Total	72	39.12	13.33	1.57	35.99	42.25	14.00	62.00

Table 4 depicts that the Sig value is less than .00; accordingly, one can conclude that there is a noticeable difference between the test anxiety post-tests of all three groups. They were at different anxiety levels at the end of the research. It seems that the experimental groups outdid the control group on the post-test.

**Table 4:** Inferential statistics of all groups on the test anxiety post-tests

	Sum of squares	Df	Mean squares	*F*	Sig.
Between groups	4635.08	2	2317.54	20.02	.00
Within groups	7986.79	69	115.75		
Total	12,621.87	71			

In Table 5, the test anxiety level of all groups is compared. This table shows that there are remarkable differences between the anxiety post-tests of the control group and both experimental groups. Also, this table shows that the formative group outdid the control and summative groups. The formative group had the best performance among the three groups of this study.

**Table 5:** Multiple comparisons by Bonferroni test (test anxiety)

(I) groups	(J) groups	Mean differences (I-J)	Std. errors	Sig.	95% confidence intervals	
					Lower bounds	Upper bounds
Control	Summative	−7.95^a^	3.10	.03	−15.57	−.33
	Formative	−19.54^a^	3.10	.00	−27.16	−11.92
Summative	Control	7.95^a^	3.10	.03	.33	15.57
	Formative	−11.58^a^	3.10	.00	−19.20	−3.96
Formative	Control	19.54^a^	3.10	.00	11.92	27.16
	Summative	11.58^a^	3.10	.00	3.96	19.20

^a^ The mean differences are significant at the 0.05 level

As observed in Table 6, all three groups’ performances on the self-regulation pre-tests are almost the same; their mean scores are almost equal. We used a one-way ANOVA to check the groups’ performances on the self-regulation pre-tests.

**Table 6:** Descriptive statistics of the three groups on the self-regulation pre-tests

	*N*	Means	Std. deviations	Std. errors	95% confidence interval for means		Minimum	Maximum
					Lower bounds	Upper bounds		
Control	24	77.54	17.02	3.47	70.35	84.73	39.00	99.00
Summative	24	78.20	16.22	3.31	71.35	85.06	41.00	101.00
Formative	24	76.83	16.78	3.42	69.74	83.92	39.00	98.00
Total	72	77.52	16.45	1.93	73.66	81.39	39.00	101.00

In Table 7, the inferential statistics of all groups on the self-regulation pre-tests are shown. As Sig (.96) is higher than (0.05), the differences between the three groups are not meaningfully significant. Based on this table, all three groups had the same level of self-regulation ability at the outset of the study.

**Table 7:** Inferential statistics of the three groups on the self-regulation pre-tests

	Sum of squares	df	Mean squares	*F*	Sig.
Between groups	22.69	2	11.34	.04	.96
Within groups	19,203.25	69	278.30		
Total	19,225.94	71			

The mean scores of the control group, the summative group, and the formative group are, 80.12, 130.04, and 147.25, respectively (Table 8). At the first look, we can say that both experimental participants outflank the control participants since their mean scores are very higher than the mean score of the control group.

**Table 8:** Descriptive statistics of the three groups on the self-regulation post-tests

	*N*	Means	Std. deviations	Std. errors	95% confidence interval for mean		Minimum	Maximum
					Lower bounds	Upper bounds		
Control	24	80.12	17.14	3.500	72.88	87.36	47.00	114.00
Summative	24	130.04	10.44	2.13	125.62	134.45	109.00	146.00
Formative	24	147.25	27.19	5.55	135.76	158.73	39.00	167.00
Total	72	119.13	34.52	4.06	111.02	127.25	39.00	167.00

The results indicate significant differences between the self-regulation post-tests of the groups in favor of the experimental groups (Table 9)*.* Based on the inferential statistics, the performances of the three groups on the self-regulation post-test are different and the summative group and the formative group outflank the control group.

**Table 9:** Inferential statistics of the three groups on the self-regulation post-tests

	Sum of square	Df	Mean squares	*F*	Sig.
Between groups	58,348.52	2	29,174.26	76.60	.00
Within groups	26,278.08	69	380.84		
Total	84,626.61	71			

The outcomes in Table 10 indicate that both experimental groups have better performances than the control group on the self-regulation post-tests. Also, the findings show that the formative group performed better than the other two groups. The treatment had the most effect on the formative group.

**Table 10:** Multiple comparisons by Bonferroni test (self-regulation)

(I) groups	(J) groups	Mean differences (I-J)	Std. errors	Sig.	95% confidence intervals	
					Lower bounds	Upper bounds
Control	Summative	−49.91^a^	5.63	.00	−63.73	−36.09
	Formative	−67.12^a^	5.63	.00	−80.94	−53.30
Summative	Control	49.91^a^	5.63	.00	36.09	63.73
	Formative	−17.20^a^	5.63	.01	−31.03	−3.38
Formative	Control	67.12^a^	5.63	.00	53.30	80.94
	Summative	17.20^a^	5.63	.01	3.38	31.03

^a^The mean differences are significant at the 0.05 level

The control group’s mean score is 90.33, the mean score of the summative group is 91.75, and the mean score of the formative group is 92.45 (Table 11). Accordingly, we can say that the three groups had an equal degree of motivation before conducting the treatment.

**Table 11:** Descriptive statistics of the three groups on the motivation pre-tests

	*N*	Means	Std. deviations	Std. errors	95% confidence interval for means		Minimum	Maximum
					Lower bounds	Upper bounds		
Control	24	90.33	25.08	5.11	79.74	100.92	50.00	149.00
Summative	24	91.75	22.08	4.50	82.42	101.07	55.00	128.00
Formative	24	92.45	21.69	4.42	83.29	101.62	55.00	129.00
Total	72	91.51	22.69	2.67	86.18	96.84	50.00	149.00

Table 12 presents the inferential statistics of all groups on the motivation pre-tests. One can see that Sig (.94) is larger than 0.50; consequently, no difference is observed among the groups in terms of motivation pre-tests. The inferential statistics show that the students of the three groups had the same amount of motivation before they had received the treatment.

**Table 12:** Inferential statistics of the three groups on the motivation pre-tests

	Sum of square	df	Mean squares	*F*	Sig.
Between groups	56.19	2	28.09	.05	.94
Within groups	36,519.79	69	529.27		
Total	36,575.98	71			

As shown in the Table 13, the mean scores of the summative and formative groups are 115.79 and 127.83, respectively, on the motivation post-tests and the mean of the control group is 92.87. It appears that the experimental participants outperform the control participants on the motivation post-tests as their mean scores are higher than the control group.

**Table 13:** Descriptive statistics of the three groups on the motivation post-tests

	*N*	Means	Std. deviations	Std. errors	95% confidence interval for means		Minimum	Maximum
					Lower bounds	Upper bounds		
Control	24	92.87	20.99	4.28	84.00	101.74	60.00	129.00
Summative	24	115.79	13.50	2.75	110.09	121.49	99.00	140.00
Formative	24	127.83	12.51	2.55	122.54	133.11	100.00	150.00
Total	72	112.16	21.58	2.54	107.09	117.23	60.00	150.00

In Table 14, the inferential statistics of all groups on the motivation post-tests are revealed. The Sig value (.00) is less than 0.50; therefore, the differences between the groups are significant. Indeed, the experimental groups outperformed the control group after the instruction and this betterment can be ascribed to the treatment.

**Table 14:** Inferential statistics of the three groups on the motivation post-tests

	Sum of square	df	Mean squares	*F*	Sig.
Between groups	15,138.08	2	7569.04	29.12	.00
Within groups	17,933.91	69	259.91		
Total	33,072.00	71			

The mean scores of the motivation post-tests are compared in Table 15. Accordingly, there are noticeable differences between the post-tests of all groups. The formative participants had better performance than the other two groups. We can say that the formative assessment is more effective than the summative assessment in EFL classes.

**Table 15:** Multiple comparisons by Bonferroni test (motivation)

(I) groups	(J) groups	Mean differences (I-J)	Std. errors	Sig.	95% confidence intervals	
					Lower bounds	Upper bounds
Control	Summative	−22.91^a^	4.65	.00	−34.33	−11.49
	Formative	−34.95^a^	4.65	.00	−46.37	−23.53
Summative	Control	22.91^a^	4.65	.00	11.49	34.33
	Formative	−12.04^a^	4.65	.03	−23.46	−.62
Formative	Control	34.95^a^	4.65	.00	23.53	46.37
	Summative	12.04^a^	4.65	.03	.62	23.46

^a^The mean differences are significant at the 0.05 level

As depicted in Table 16, the amount of statistic *T*-value is 63.72, *df*=16, and *Sig*=0.00 which is less than 0.05. This implies that Iranian students held positive attitudes towards the effectiveness of summative and formative assessments on their language learning improvement.

**Table 16:** One-sample test of the attitude questionnaire

	Test value = 0					
	*T*	Df	Sig. (2-tailed)	Mean differences	95% confidence interval of the differences	
					Lower	Upper
Scores	63.72	16	.000	4.52	4.37	4.67

Briefly, the results indicate that both experimental groups had better performances than the control group in their post-tests. The formative group had the best performance among the three groups of this study. Additionally, the results reveal that the participants of the present research had positive attitudes towards the effectiveness of both formative and summative assessments on their language learning development.

After analyzing the data, it was found that all three groups were at the same levels of test anxiety, motivation, and self-regulation skill at the outset of the research. But, the performances of the three groups were different at the end of the investigation. Both experimental groups outdid the control group on their post-tests and the formative group performed better among the three groups. Although both types of assessments (summative and formative) were effective on test the anxiety, motivation, and self-regulation skill of EFL learners, the formative assessment was the most effective one. The findings of the current research also indicated that both experimental groups presented positive attitudes toward the implementation of the summative and formative assessments in EFL classes.

The findings gained in this study are supported by Persaud Singh and Ewert (2021) who inspected the impacts of formative assessment on adult students’ language improvement. They indicated that there were meaningful differences between the formative participants and the control participants in terms of language achievement in favor of the formative participants. Additionally, our research findings are advocated by Alahmadi et al. (2019) who explored the effects of formative speaking assessments on EFL learners’ performances in speaking tests. They showed that the formative assessment assisted Saudi EFL learners to solve the problems they encountered in speaking tests.

In addition, our study findings are in accordance with Mahshanian et al. (2019) who confirmed that the amalgamation of summative and formative assessment can result in better achievement in English language learning. Also, our investigation lends support to the findings of Buyukkarci and Sahinkarakas (2021) who verified the positive effects of using formative assessment on learners’ language achievement. Additionally, the results of the current research are in agreement with Ounis (2017) who stated that formative assessment facilitated and supported students’ learning. Our study findings are supported by the sociocultural theory which focuses on the role of social interactions among the students and their teachers in the classroom. Based on this perspective, the learning process is mainly a social process and students’ cognitive functions are made based on their interactions with those around them.

Furthermore, our research results are in agreement with the results of Imen (2020) who discovered the impacts of formative assessments on EFL students’ writing abilities. His results indicated that using formative assessment develops the participants’ writing skills. Moreover, our research outcomes are supported by the impacts of formative assessments on learners’ academic attainment, opinions about lessons, and self-regulation skills in Ozan and Kıncal (2018) who performed an investigation on the influences of formative assessments on students’ attitudes toward lessons, academic achievement, and self-regulation skill. They revealed that the experimental class that received the treatment by formative assessment practices had better academic performances and more positive attitudes towards the classes than the control class.

Regarding the positive attitudes of the participants towards formative and summative assessment, our results are in line with Tekin (2010) who discovered that formative assessment practices meaningfully developed students’ attitudes about mathematics learning. That research indicated that the participants in the treatment group had positive attitudes about mathematics learning. In addition, King (2003) asserted that the formative assessments enhanced the learners’ attitudes about science classes. Also, Hwang and Chang (2011) revealed that the formative assessment highly boosted the attitudes and interest of students toward learning in local culture classes.

One explanation for the outperformance of the formative group over the other two groups can be the fact that they received much more input. They were provided with different kinds of feedback and took more exams during the semester. These exams and feedback can be the reasons for their successes in language achievement. This is in line with Krashen’s (1981) input theory stating that if students are exposed to more input, they can learn more.

The other possible explanations for our results are that formative assessments are not graded so they take the anxiety away from the assessees. They also detach the thinking that they must get everything right. Instead, they serve as a practice for students to get assistance along the way before the final tests. Teachers usually check for understanding if students are struggling during the lesson. Teachers address these issues early on instead of waiting until the end of the unit to assess. Teachers have to do less reteaching at the end because many of the problems with mastery are addressed before final tests. The mentioned advantages can be the reasons for our obtained findings.

In addition, monitoring the students’ learning via using the formative assessment can be the other justification for our results. In fact, monitoring the learning process can provide an opportunity for the teachers to give constructive feedback to their students to improve their language learning. When teachers continuously monitor students’ growth and modify instruction to ensure constant development, they find it easier and more predictable to progress towards meeting the standards on summative assessments. By comprehending precisely what their students know before and during the instruction, teachers have much more power to improve the students’ mastery of the subject matter than if they find out after a lesson or unit is complete.

It is important to point out that when instructors continually evaluate the development of their students and modify their curriculum to assure constant improvement, they find that it is simpler and more predictable to make progress toward fulfilling the requirements on summative assessments. If teachers wait until the end of a session or unit to find out how well their learners have mastered the material, they will have considerably less influence over how well their learners learn the material than if they find out how well their learners have mastered it earlier and during teaching. The value of formative assessment lies in the critical information about student comprehension that it provides throughout the process of learning, as well as the chance it gives educators to provide participants with quick and efficient, and action-oriented feedback, as well as the chance to alter their own behavior so that every respondent has the chance to learn and re-learn the material. Learners whose academic performance falls on the extreme ends of the normal curve, such as those who are struggling and those who excel academically, benefit the most from formative evaluation. These learners have learning requirements that are often one of a kind and highly specialized, and to meet those needs, the instructor needs updated data. In addition, making use of frequent formative evaluation as a means to remediate learning gaps brought up by COVID-19 guarantees that educators can promptly give remediation.

The other justification for our findings can be ascribed to the strength of formative assessments that lies in the formative information they provide about the students’ comprehension throughout the learning process and the opportunities they give to teachers to provide the pupils with action-oriented and timely feedback and to change their own behaviors so that each learner has an opportunity to learn and re-learn. More particularly, using formative assessment can assist the students to detect their own weaknesses and strengths and target areas that need more effort and work. All the positive points enumerated for the formative assessments can be the reasons and explanations for the results gained in the current research.

Moreover, the better performance of assessment groups may be due to numerous reasons. In the first place, consistently evaluating students’ progress helps maintain learning objectives at the forefront of one’s mind. This ensures that learners have a distinct goal to strive towards and that instructors have the opportunity to assist clear up misconceptions before learners get off track. Second, engaging in the process of formative assessment enables instructors to gather the information that reveals the requirements of their students. When instructors have a clear grasp of what it takes for their students to be successful, they are better able to design challenging educational environments that push every learner to their full potential. Thirdly, the primary role of formative assessment that will assist in enhancing academic achievement is to provide both learners and instructors with frequent feedback on the achievement that is being made toward their objectives. Learners can bridge the gap between their existing knowledge and their learning objectives through the use of formative assessment (Greensetin, 2010). The fourth benefit of doing the formative assessment is an increase in motivation. Formative assessment entails creating learning objectives and monitoring the progress towards those objectives. When learners have a clear idea of where they want to go, their performance dramatically improves. Fifthly, students must identify a purpose for the work that is assigned to them in the classroom. Connecting the learning objectives with real-world problems and situations draws students into the instructional activities and feeds their natural curiosity about the world. Sixthly, an in-depth examination of the data gathered via formative assessment provides the educator with the opportunity to investigate their own methods of teaching and identify those that are successful and those that are not. It is indeed possible that some of the strategies that work for one group of learners won’t work for another. Lastly, students become self-regulated when they are provided with the tools they need to set, track, and ultimately achieve their own learning objectives. Students may develop into self-reliant thinkers if they are exposed to models of high-quality work and given adequate time to reflect on and refine their own work.

The positive effects of formative and summative assessment on students’ motivation are supported by The Self Determination Theory (SDT) of Motivation which is a motivational theory that provides a way of understanding human motivation in any context (Ryan & Deci, 2000). SDT attempts to understand human motivation beyond the simple intrinsic/extrinsic model. It suggests that human motivation varies from fully intrinsic motivation, which is characterized by fully autonomous behavior and “for its own sake” to fully extrinsic motivation, which is characterized by behavior that is fully heteronomous and which is instrumentalized to some other end.

In this study, the self-regulatory skills of the students in the EGs where the formative assessment practices were applied did significantly differ from the ones in the CG where no formative assessment practices were applied. Thus, students’ self-regulation was shown to be improved as a result of formative assessment procedures. Similar findings were observed in the experimental research by Xiao and Yang (2019) that compared the self-regulation abilities of EG and CG learners in secondary school and discovered a substantial difference in favor of the former group. Research findings based on qualitative data reveal that learners engaged in a variety of cognitive techniques and self-regulatory learning practices. The participants acknowledged that they were an integral part of their own learning and that they accepted personal responsibility for their progress. Teachers reported that learners’ ability to self-regulate improved as a result of formative assessment, which fostered ongoing, meaningful, and learning-effort and performance-focused dialogue between teachers and learners. The students’ progress in the areas of self-regulation and metacognitive abilities, as well as their growth in accordance with educational standards, may be supported by a rise in their success in diagnostic examinations thanks to the use of formative assessment (DeLuca et al., 2015). In a study that he conducted in 2015, Woods examined the link between formative assessment and self-regulation. He highlighted that teachers who use formative assessment strategies need to comprehend the participants’ self-regulatory learning processes to make appropriate decisions for their classrooms. Furthermore, Woods (2015) recommended that educators make regular use of formative assessment to foster the growth of learners’ abilities to self-regulate and to boost the motivation levels of their learners. Wiliam (2014) also asserted that self-regulatory learning could be an important component of an effective formative assessment in relation to the techniques of explaining, sharing, and comprehending the learning goals and success criteria and students taking the responsibility for their own learning.

It is vital to note that learners who have developed self-regulation skills employ their cognitive abilities; work toward their learning objectives; seek out appropriate support from peers, adults, and authority figures; and, most significantly, accept personal accountability for their academic success. As a result, learners’ abilities to self-regulate have a direct effect on the type of formative assessment based on learning and the applications designed to eliminate learning deficiencies. Self-regulation is an ability that needs time and practice to acquire, but it is possible to do so with the right tools and a continuous strategy. Formative assessment techniques were shown to boost learners’ ability to self-regulate, although this effect was found to be small when the study findings were combined with those found in the literature. This finding may be attributed to the fact that, although formative assessment procedures were implemented for an academic year, they were limited to the context of the social research classroom, and students’ abilities to self-regulate may develop and evolve over time.

The findings of this research can increase the knowledge of the students about two types of assessment. This study can encourage students to want their teachers to assess their performances formatively during the semester. Also, the findings of this study can assist instructors to implement more formative-based assessments and feedback in their classes. This study can highlight the importance of frequent input, feedback, and exam for teachers. An exact analysis of formative assessment data permits the teachers to inspect their instructional practices in order to understand which are producing positive results and which are not. Some that are effective for one group of students may not be effective for another group. The implications of this research can help students try to compensate for their deficiencies by taking responsibility for their own learning instead of just attempting to get good grades. In this respect, formative assessments ensure that students can manage the negative variables such as a high level of examination and grading.

Using formative assessments helps teachers gather the information that reveals the students’ needs. Once teachers have an understanding of what students need to be successful, they can generate a suitable learning setting that will challenge each learner to grow. Providing students and teachers with regular feedback on progress towards their aims is the major function of the formative assessments that will help in increasing academic accomplishment. Formative assessments can help the students close the gap between their present knowledge and their learning objectives. Moreover, using formative assessment gives the students evidence of their present progress to actively monitor and modify their own learning. This also provides the students the ability to track their educational objectives. Also, via using formative assessment, the students have the ability to measure their learning at a metacognitive level. As the students are one of the main agents of the teaching-learning process, instructors must share the learning objectives with them. This sharing can develop the students’ learning in basic knowledge and higher order cognitive processes such as application and transfer (Fulmer, 2017). In fact, if learners know that they are expected to learn in that lesson, they will concentrate more on those areas. Formative assessments make the teaching more effective by guiding learners to achieve learning objectives, setting learning needs, modifying teaching accordingly, and increasing teachers’ awareness of efficient teaching methods. Lastly, our findings may aid material developers to implement more formative-based assessment activities in the EFL English books.

## Conclusion

In conclusion, this study proved the positive impacts of applying formative assessments on Iranian EFL students’ academic motivation, attitude toward learning, test anxiety, and self-regulation skill. Therefore, teachers are strongly recommended to use formative assessment in their classes to help students improve their language learning. Using formative assessment allows teachers to modify instruction according to the results; consequently, making modifications and improvements can generate immediate benefits for their students’ learning.

One more conclusion is that using formative assessment gives the teacher the ability to provide continuous feedback to their students. This allows the students to be part of the learning environment and to improve self-assessment strategies that will help with the understanding of their own thinking processes. All in all, providing frequent feedback during the learning process is regarded as an efficient technique for motivating and encouraging students to learn a language more successfully. Indeed, by assessing students during the lesson, the teachers can aid them to improve their skills and examine if they are progressing or not. Thus, formative assessment is an essential part of teaching that should be used in EFL instructional contexts.

As we could not include many participants in our study, we recommend that future researchers include a large number of participants to increase the generalizability of their results. We worked on male EFL learners; the next studies are required to work on both genders. We could not gather qualitative data to enrich our results; the upcoming researchers are advised to collect both quantitative and qualitative data to develop the validity of their results. Next researchers are called to examine the effects of the summative and formative assessments on language skills and sub-skills. Also, next researchers are offered to inspect the effects of other types of assessments on language skills and subskills as well as on psychological variables involved in language learning.

## Data Availability

The data that support the findings of this study are available from the corresponding author upon reasonable request.

## Abbreviations

EFL: English as a foreign language
ANOVA: Analysis of variance
PET: Preliminary English Test
SAS: Science Anxiety Scale
SRSS: Self-Regulatory Strategies Scale
AMTB: Attitude/Motivation Test Battery
SDT: Self Determination Theory
EG: Experimental group
CG: Control group
